# Preconditioning with Physiological Levels of Ethanol Protect Kidney against Ischemia/Reperfusion Injury by Modulating Oxidative Stress

**DOI:** 10.1371/journal.pone.0025811

**Published:** 2011-10-12

**Authors:** Qing Yuan, Shanjuan Hong, Shu Han, Li Zeng, Fang Liu, Guoshan Ding, Yindong Kang, Jingyan Mao, Ming Cai, Youhua Zhu, Quan-xing Wang

**Affiliations:** 1 Organ Transplantation Institute of PLA, Changzheng Hospital, Second Military Medical University, Shanghai, China; 2 National Key Laboratory of Medical Immunology, Second Military Medical University, Shanghai, China; 3 Organ Transplant Center, The 309th Hospital of PLA, Beijing, China; Universidade de Sao Paulo, Brazil

## Abstract

**Background:**

Oxidative stress due to excessive production of reactive oxygen species (ROS) and subsequent lipid peroxidation plays a critical role in renal ischemia/reperfusion (IR) injury. The purpose of current study is to demonstrate the effect of antecedent ethanol exposure on IR-induced renal injury by modulation of oxidative stress.

**Materials and Methods:**

Bilateral renal warm IR was induced in male C57BL/6 mice after ethanol or saline administration. Blood ethanol concentration, kidney function, histological damage, inflammatory infiltration, cytokine production, oxidative stress, antioxidant capacity and Aldehyde dehydrogenase (ALDH) enzymatic activity were assessed to evaluate the impact of antecedent ethanol exposure on IR-induced renal injury.

**Results:**

After bilateral kidney ischemia, mice preconditioned with physiological levels of ethanol displayed significantly preserved renal function along with less histological tubular damage as manifested by the reduced inflammatory infiltration and cytokine production. Mechanistic studies revealed that precondition of mice with physiological levels of ethanol 3 h before IR induction enhanced antioxidant capacity characterized by significantly higher superoxidase dismutase (SOD) activities. Our studies further demonstrated that ethanol pretreatment specifically increased ALDH2 activity, which then suppressed lipid peroxidation by promoting the detoxification of Malondialdehyde (MDA) and 4-hydroxynonenal (HNE).

**Conclusions:**

Our results provide first line of evidence indicating that antecedent ethanol exposure can provide protection for kidneys against IR-induced injury by enhancing antioxidant capacity and preventing lipid peroxidation. Therefore, ethanol precondition and ectopic ALDH2 activation could be potential therapeutic approaches to prevent renal IR injury relevant to various clinical conditions.

## Introduction

Ischemia/reperfusion (IR), a common pathological process, occurs under various clinical conditions such as trauma, hypovolemic shock, sepsis, and most importantly renal transplantation. Surgical procedures requiring cross clamping of the aorta and renal vessels are associated with a renal failure rate up to 30% [1], and only a mild increase in the serum creatinine levels (0.3–0.4 mg/dl) is associated with a 70% greater risk of death [2]. In renal transplantation, the IR injury not only contributes to the early functional failure or delayed function for the grafts, but also strongly increases the incidence of both acute and chronic rejection, and therefore, it dampens the long-term graft survival. Given the fact that organ shortage has forced the compromise of using organs from expanded criteria of donors, which renders the renal grafts subject to more severely IR injury [3]. Therefore, the need for additional therapeutic modalities to prevent renal IR injury is quite urgent.

The pathophysiology of renal IR injury includes both direct cellular damage caused by ischemic insult, and delayed dysfunction/damage due to the activation of inflammatory pathways [4]. Since inflammatory response is a sequential event, it would be more relevant to block the initial signal [5], [6], [7], [8]. Oxidative stress, a common event caused by excessive production of reactive oxygen species (ROS) during IR process, is a critical factor implicated in both direct and subsequent cellular damage [9], [10]. Indeed, antioxidant therapy has already been shown to be protective against IR-mediated oxidative damage in different experimental models [11], [12]. Lipid peroxidation acts as one of the most important sources of oxidative stress, and previous studies have already demonstrated evidence indicating its implication in renal IR injury [13], [14]. It is an autocatalytic mechanism resulting in oxidative destruction of cellular membranes associated with the production of toxic, reactive aldehydic metabolites and cell death [15]. Malondialdehyde (MDA) and 4-hydroxynonenal (HNE) are the most important aldehydic metabolites. Excessive production of these highly cytotoxic metabolites would cause their diffusion from the site of origin to attack distant targets, through which they form covalent links with various molecules (adducts) to mediate the inactivation of enzymes and inhibition of DNA, RNA and protein syntheses.

The deleterious effects of lipid peroxidation might be prevented by enhancing the scavenging systems. Indeed, studies in cardiac ischemia suggested that pretreatment with physiological levels of ethanol can provide cardioprotection, which is consistently correlated with the phosphorylation status of Aldehyde Dehydrogenase 2 (ALDH2), a key metabolic enzyme in the oxidation and detoxification of reactive aldehydes in a range of organs and cell types [16], [17]. ALDH2 is a mitochondrial enzyme, belonging to the ALDH gene family. ALDH2 not only catalyzes the oxidation of acetaldehyde to acetic acid in ethanol metabolism, but also acts as a key metabolic enzyme involved in the detoxification of other reactive aldehydes such as HNE [18]. Other than cardiac IR injury, the protective effect of antecedent ethanol exposure has also been proved in the brain and intestine IR injury [19], [20], [21]. However, virtually no information is available regarding to its role in IR-induced renal damage. Given the fact that consumption of red wine at low to moderate levels is beneficial for renal disease and ethanol pretreatment prevents postischemic leukocyte-endothelial cell adhesive interactions that is critical for inflammatory infiltration after IR, we therefore hypothesized that pretreatment with physiological levels of ethanol would protect kidneys against IR injury by modulating antioxidant capability and preventing lipid peroxidation.

## Materials and Methods

### Animals and ethics statement

Male C57BL/6 mice (8–12 wk, weight 20–25 g) were obtained from Joint Ventures Sipper BK Experimental Animal (Shanghai, China). All animal experiments were performed in accordance with the guidelines of National Institute of Health for the Care and Use of Laboratory Animals, and approved by the Scientific Investigation Board of Second Military Medical University, Shanghai, China.

### Induction of renal IR

Mice were anesthetized with sodium pentobarbital (*i.p.*, 50 mg/kg). After a midline laparotomy, renal pedicles on both sides were closed for 30 min with nontraumatic microaneurysm clamps (Shanghai Medical Instruments, Shanghai, China). The incision was temporarily closed during ischemia. The abdomen was closed after visual verification of reperfusion upon the removal of microaneurysm clamps. Mice with sham surgery (no interruption of the renal blood flow) were used as controls. Body temperature was maintained with an adjustable heating pad at 37°C. All mice were *i.p.* injected with 500 µl 0.9% NaCl upon completion of the surgery, and were sacrificed 24 h or day 7 after reperfusion. At the time of sacrifice, blood was collected through inferior vena cava into a heparinized syringe for plasma biochemistry within 24 h. Kidneys were fixed in 10% neutral-buffered formalin for paraffin embedding or snap frozen for later use. Some mice were housed individually in metabolism cages in the first 24 h after reperfusion for collection of urine samples. For ethanol pretreatment, mice were injected different dose of ethanol (0.25, 0.5, 1, 2 g/kg body weight) before the induction of kidney IR. The control group received the largest volume of saline as that in the 2 g/kg body weight dose group.

### Primary culture of mouse renal tubular epithelial cells and simulated IR

Primary mouse renal tubular epithelial cells (TEC) were cultured following a method described by Wuthrich et al. [22]. Briefly, kidneys were flushed *in situ* with 0.9% NaCl to remove blood. The outer cortex was cut into pieces of approximately 1 mm^3^, and digested in HBSS containing 1 mg/ml of collagenase type 1 (Worthington) at 37°C for 30 min and washed through a sieve (mesh diameter 75 µm) in DMEM/F12 medium (Invitrogen) to obtain single cell suspensions. After centrifugation at 300 g for 5 min, the pellet was resuspended in DMEM/F12 medium supplemented with 1% penicillin/streptomycin and 10% FCS (Invitrogen), and incubated at 37°C for 3 h in a Petri dish to allow contaminated glomerular cells to adhere. The non-adherent tubular cells were next collected and cultured in 6 - well plates in DMEM/F12 medium supplemented with 10% FCS until epithelial colonies were established. TECs were identified by cobblestone-shaped morphology and expression of cytokeratin 18 (CK18) using an anti-CK18 antibody (Santa Cruz) as described by Wu et al. [23]. Tubular cells prepared as above showed >95% of purity.

IR was simulated by immersing cell monolayer in mineral oil as previously described [24]. In brief, TECs were starved in serum-free (SF) DMEM/F12 medium for 24 h. After washes with PBS, the cells were immersed in mineral oil (Sigma-Aldrich) for 60 min at 37°C. After extensive washes with PBS, the cells were incubated in DMEM/F12 medium supplemented with 10% FCS, and subjected to functional analysis 24 h after medium replacement.

### Assessment of renal function

Plasma creatinine (P_Cr_), urea, sodium (P_Na_), urine creatinine (U_Cr_) and sodium (U_Na_) were assessed using a standard autoanalyzer at the Central Laboratory, Changzheng Hospital, Shanghai. Urinary osmolality (U_Osm_) was measured using an automatic freezing point osmometer (L1590031, Xiyu Electrical and Mechanical Systems, Inc., Shanghai, China). Fractional excretion of sodium (FE_Na_, %) was calculated from the standard formula FE_Na_ = P_Cr_×U_Na_×100/(U_Cr_×P_Na_).

### Measurement of oxidation

Thiobarbituric acid-reactive substances (Sigma) were employed to assess lipid peroxidation. Samples were evaluated for MDA production via a spectrophotometric assay for thiobarbituric acid-reactive substances [25], [26]. The amount of SOD activity from renal homogenates was measured with a commercial SOD assay kit (Cayman Chemical, Ann Arbor, MI). Hydroxynonenal (HNE) concentration was determined using fresh cytosolic fractions with the method described by Esterbauer et al [27]. An aliquot of cytosol (200 µl) was extracted using equal volume of acetic acid/acetonitrile solution (4 : 96, vol : vol). After centrifugation at 250 g for 20 min at 4°C, 50 µl of supernatant were injected into a high-performance liquid chromatography (HPLC) Symmetry C18 column (5 mm, 3.9×150 mm). The mobile phase used was acetonitrile:bidistilled water (42%, vol : vol). The HNE concentration was calculated based on a standard concentration curve (Calbiochem-Novabiochem Corp., La Jolla, CA, USA).

### Histological examination

Kidneys embedded in paraffin were sectioned at 2 µm and stained with H&E by standard methods. Tubular damage was estimated using a grading scale of 0–4, as outlined by Jablonski et al. [28] Three representative sections from each kidney were scored. At least 10 high-power fields (×400) per section were examined for each sample. Histological examination was performed by two pathologists in a blinded fashion.

### Real-time RT-PCR analysis

Total kidney RNA was extracted using TRIzol (Invitrogen) reagent according to the manufacturer's instructions. cDNA was synthesized using oligo d(T) (Applied Biosystems) and a SuperScript III Reverse Transcriptase Kit (Invitrogen). A StepOne™ Real-Time PCR System (Applied Biosystems) and a SYBR RT-PCR kit (Takara) were used for quantitative real-time RT-PCR analysis. All reactions were conducted in a 20 µl reaction volume in triplicate. The relative expression levels for a target gene were normalized by *GAPDH*. Specificity was verified by melting curve analysis and agarose gel electrophoresis. Primers used RT-PCR analysis are: TNF-α (5′-AAG CCT GTA GCC CAC GTC GTA-3′; 5′-GGC ACC ACT AGT TGG TTG TCT TTG-3′); IL-6 (5′-ACA ACC ACG GCC TTC CCT ACT T-3′; 5′-CAC GAT TTC CCA GAG AAC ATG TG-3′); IL-8 (5′-ATG CCC TCT ATT CTG CCA GAT-3′; 5′-GTG CTC CGG TTG TAT AAG ATG AC-3′); IL-10 (5′-GCT CTTA CTG ACT GGC ATG AG-3′; 5′-CGC AGC TCT AGG AGC ATG TG-3′); CD3 (5′-AGA GGG CAA AAC AAG GAG CG-3′; 5′-AGA CTG CTC TCT GAT TCA GGC-3′); CD11b (5′-ATG GAC GCT GAT GGC AAT ACC-3′; 5′-TCC CCA TTC ACG TCT CCC A-3′); iNOS (5′- ACA TCG ACC CGT CCA CAG TAT -3′; 5′- CAG AGG GGT AGG CTT GTC TC-3′); eNOS (5′-TCA GCC ATC ACA GTG TTC CC-3′; 5′- ATA GCC CGC ATA GCG TAT CAG-3′); and GAPDH (5′-TGA CCA CAG TCC ATG CCA TC-3′; 5′-GAC GGA CAC ATT GGG GGT AG-3′). Data were analyzed using the comparative C_t_ (2^−ΔΔCt^) method.

### ELISA and renal MPO activity assay

For measurement of myeloperoxidase (MPO) activity in renal homogenates, kidneys were homogenized in a buffer containing 50 mM Tris–HCl, 1 mM EDTA and 5% Sucrose (pH 7.4). The detailed steps were in accordance with manufacturer's protocol of the kit (Jiancheng Bioengineering Institute, Nanjing, China). MPO activity was expressed as ΔOD per minute per milligram total protein in renal lysates.

### Immunoblot analysis

Snap-frozen renal tissues or cultured cells were lysed with the M-PER Protein Extraction Reagent (Pierce) supplemented with a protease inhibitor ‘cocktail’. The samples were incubated for 30 min at 4°C and centrifuged at 16,000 g at 4°C for 15 min. Lysates were collected and stored at −70°C until use. Protein concentration in the lysates was measured with a bicinchoninic acid assay (Pierce). Equal amount of lysates were separated by SDS-PAGE, and then transferred onto nitrocellulose membranes for immunoblot analysis.

### Measurement of ethanol concentration in the plasma

Ready-to-use Q.E.D. A150 Alcohol Test kit (OraSure Technologies, Bethlehem, PA) was used to quantify alcohol levels in the plasma samples.

### Assay for ALDH enzymatic activity

Enzymatic activity for ALDH was determined spectrophotometrically by monitoring the reductive reaction of NAD^+^ to NADH at 340 nm as previously described [16], [17], [29]. ALDH2 assays were carried out at 25°C in 50 mM sodium pyrophosphate buffer (pH = 9.5). The reaction includes 10 mM acetaldehyde and 400 µg of lysate protein isolated from kidneys. The accumulation of NADH was monitored for 5 min with measurements being taken every 30 s after addition of 2.5 mM NAD. ALDH reactive rates were expressed as µmol NADH/min/mg protein.

### Knockdown of ALDH2 in TECs

Mouse primary cultured TECs were transfected with a negative control siRNA (NC) or an ALDH2-siRNA, using the INTERFERin™ transfection reagent (Polyplus). TECs treated with the INTERFERin™ transfection reagent only were served as another negative control. The assays were conducted 48 h after transfection.

### Measurement of lactate dehydrogenase release

Lactate dehydrogenase (LDH) in the culture medium and primary renal TECs was measured using the modified colorimetric routine laboratory method [30]. Briefly, culture supernatants or cell lysates were incubated with 0.2 mmol/l β-NADH and 0.4 mmol/l pyruvic acid diluted in PBS (pH 7.4). It was also confirmed that addition of ethanol to the incubation mixture did not alter the oxidation of β-NADH. LDH concentration in the sample was proportional to the linear decrease in the absorbance at 334 nm, and calculated using a commercial standard. The percentage of LDH released from primary renal TECs was calculated as LDH present in culture supernatant in relation to total LDH obtained in the culture medium and primary renal TECs.

### Statistics

Data from multiple groups were analyzed using one way ANOVA with post-hoc Bonferroni's correction (GraphPad Prism 5.0; GraphPad Software). Data derived from two groups were analyzed using an unpaired Student's *t* test or a Mann - Whitney test (two tailed). All data were expressed as mean ± SEM. In all cases, *p*<0.05 was considered with statistical significance.

## Results

### 1. Blood ethanol concentration

**Figure 1:**
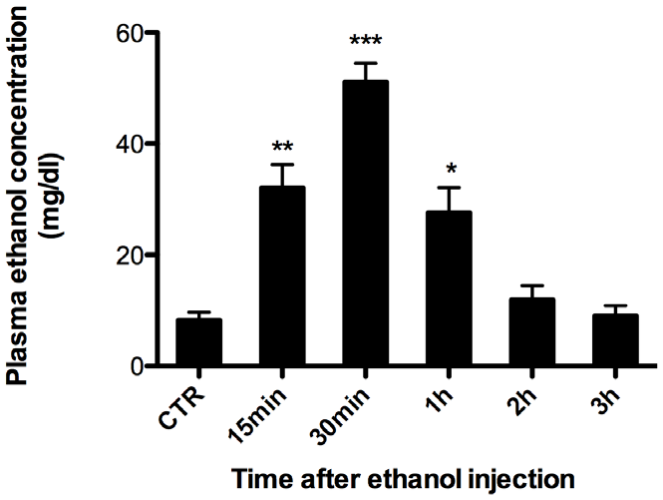
Plasma ethanol concentrations in mice after ethanol administration. Mice received an *i.p.* injection of 5% ethanol at a dose of 1 g/kg body weight were sacrificed at indicated time after, and plasma ethanol concentration were assayed. The CTR group did not receive any ethanol. Results are mean values ± SEM (*n* = 4–6). (* *p*<0.05, ** *p*<0.01, *** *p*<0.001; in comparison with the CTR group).

We first monitored the temporal blood ethanol concentration after intra-peritoneal (*i. p.*) injection. To this end, the mice were *i.p.* injected with 5% ethanol at a dose of 1 g/kg body weight. The mice were next sacrificed at indicated time point and ethanol concentration in the plasma was determined as described. As shown in Figure 1, high levels of ethanol can be detected as early as 15 min after *i.p.* injection, and the highest level (approximately 51 mg/dl or 11 mM) was observed 30 min after injection, which is equivalent to the physiologically attainable blood ethanol levels in humans after one to three drinks [31], [32]. The blood ethanol then underwent a steady decrease and 3 h after injection returned to the level as controls. Together, our data demonstrated a similar result as that obtained using the gastric ingestion approach [19], [20].

### 2. Ethanol pretreatment protects kidneys against IR injury

**Figure 2:**
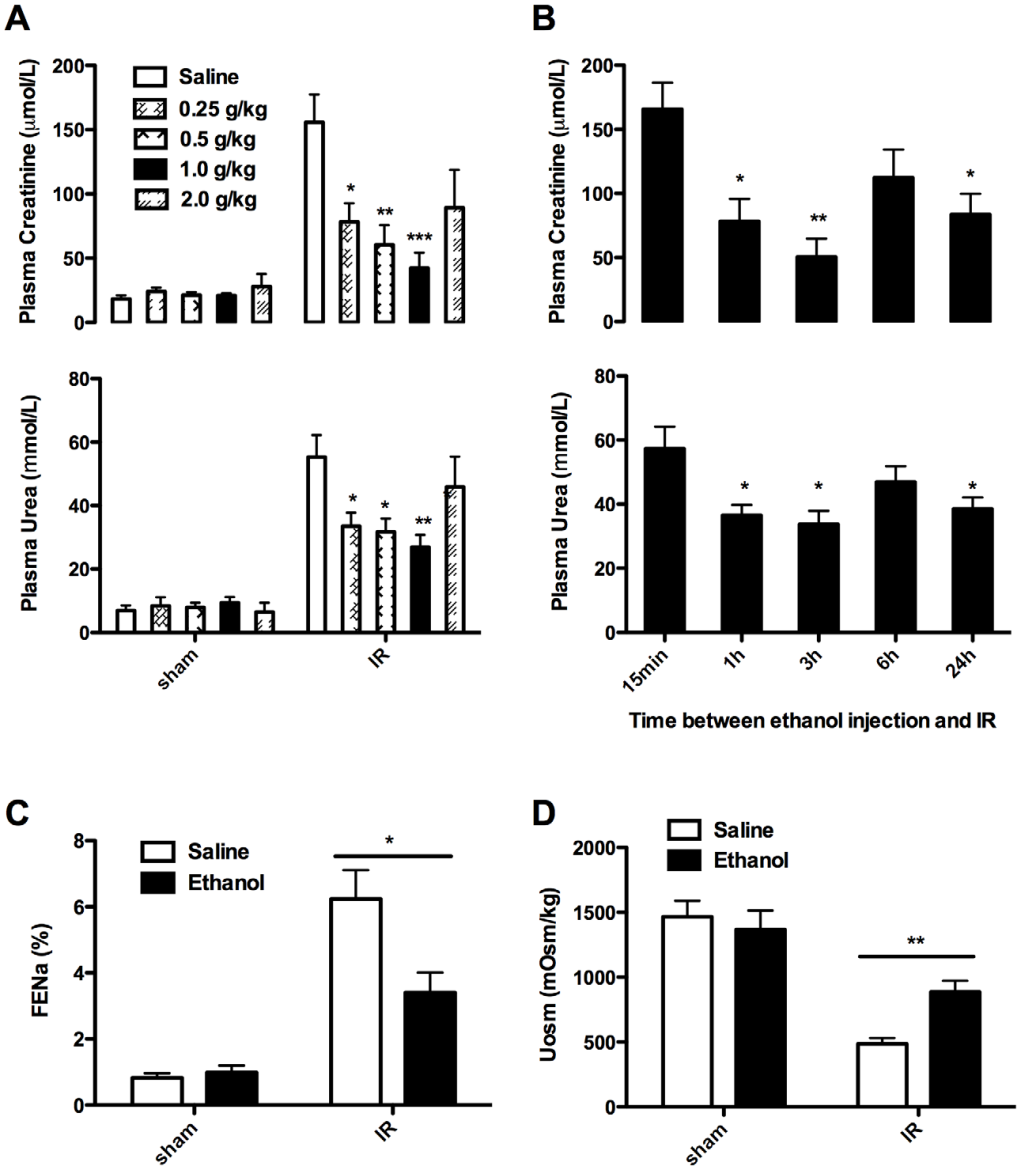
Ethanol pretreatment preserved kidney function after renal IR. **A.** Mice were pretreated with saline or ethanol at different dose. A 30 min bilateral renal ischemia was induced 3 h later. Blood samples were harvested at 24 h after reperfusion to assess the plasma creatinine and urea. Sham controls underwent the same procedure without vascular occlusion. Results are mean values ± SEM (*n* = 4–8). **B.** Mice were pretreated with ethanol (1 g/kg) and exposed to a 30 min bilateral renal ischemia after different time interval. Blood samples were harvested at 24 h after reperfusion to assess the plasma creatinine and urea. Results are mean values ± SEM (*n* = 5–6). **C. & D.** Mice were pretreated with saline or 1 g/kg ethanol, and were exposed to a 30-min bilateral renal IR 3 h later. FE_Na_ and Uosm were assessed at 24 h after reperfusion. (* *p*<0.05, ** *p*<0.01, *** *p*<0.001, in comparison with saline control of that group (sham or IR) for **A.**, and with the 15 min group for **B.**).

To assess the effect of ethanol pretreatment on renal IR injury, we administered mice with different doses of ethanol, and 3 h later the mice were subjected to induction of bilateral renal IR injury. It was interestingly noted that antecedent ethanol exposure dose-dependently protected kidneys against IR-induced injury (Figure 2A). The strongest protective effect was observed when mice administrated with 1 g/kg ethanol, in which the plasma creatinine decreased by 72.8% as compared with that of mice administrated with normal saline. However, for those doses above 1 g/kg such as 2 g/kg failed to provide a significant protective effect. Therefore, 1 g/kg is likely the most optimal dose for mediating the protective effect.

Next, we injected mice with 1 g/kg of ethanol, followed by induction of those mice with bilateral renal IR injury at indicated time point. The protective effect was noted as early as 1 h after ethanol administration and lasted until 24 h post ethanol administration as manifested by the lower plasma creatinine and urea (Figure 2B). Particularly, the most prominent protective effect was observed 3 h after ethanol administration, in which the amount of plasma creatinine and urea was only about 30.4% and 58.8% as that of control mice, respectively. We further assessed tubular function by measuring the fractional excretion of sodium (FE_Na_) and urine osmolality (U_Osm_). Ethanol pretreatment significantly decreased FE_Na_ but increased urine osmolality as compared with that of controls (Figure 2C & 2D).

**Figure 3:**
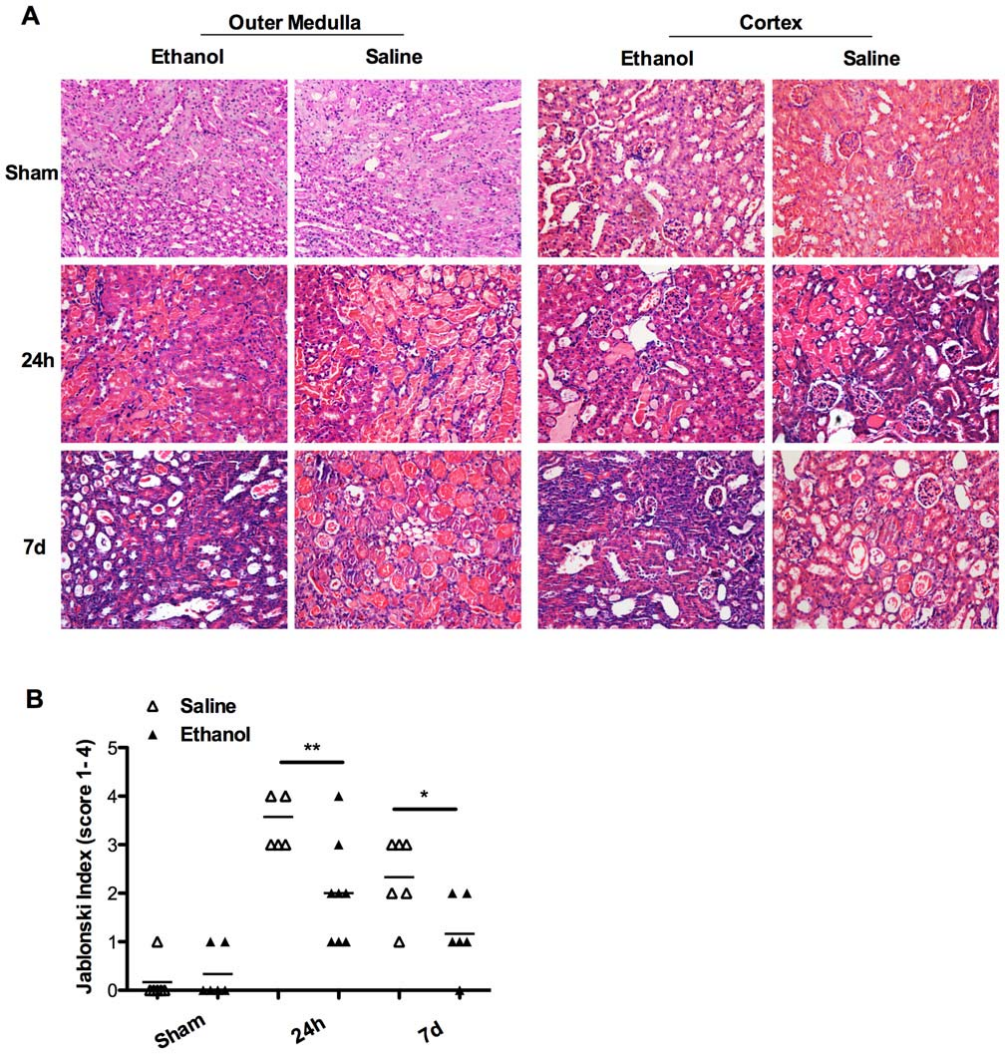
Ethanol pretreatment attenuated tubular damage after renal IR. Mice were pretreated with saline or 1 g/kg ethanol, and were exposed to a 30-min bilateral renal IR 3 h later. Kidney samples were collected at 24 h post reperfusion and assessed for: **A.** H&E staining of renal outer medulla (left panel) and cortex (right panel). Sections are representative of 6–8 independent mice per experimental group (200× original magnification). **B.** Scoring of the tubular injury according to the Jablonski's criteria. (* *p*<0.05, ** *p*<0.01).

In line with the above results, histological analysis of renal sections 24 h and day 7 after IR induction revealed that antecedent ethanol exposure reduced tubular injury as characterized by less tubular necrosis, less severity for loss of the brush border and cast formation (Figure 3A). The Jablonski index further confirmed the protective effect of antecedent ethanol exposure in IR-induced tubular damage (Figure 3B).

**Figure 4:**
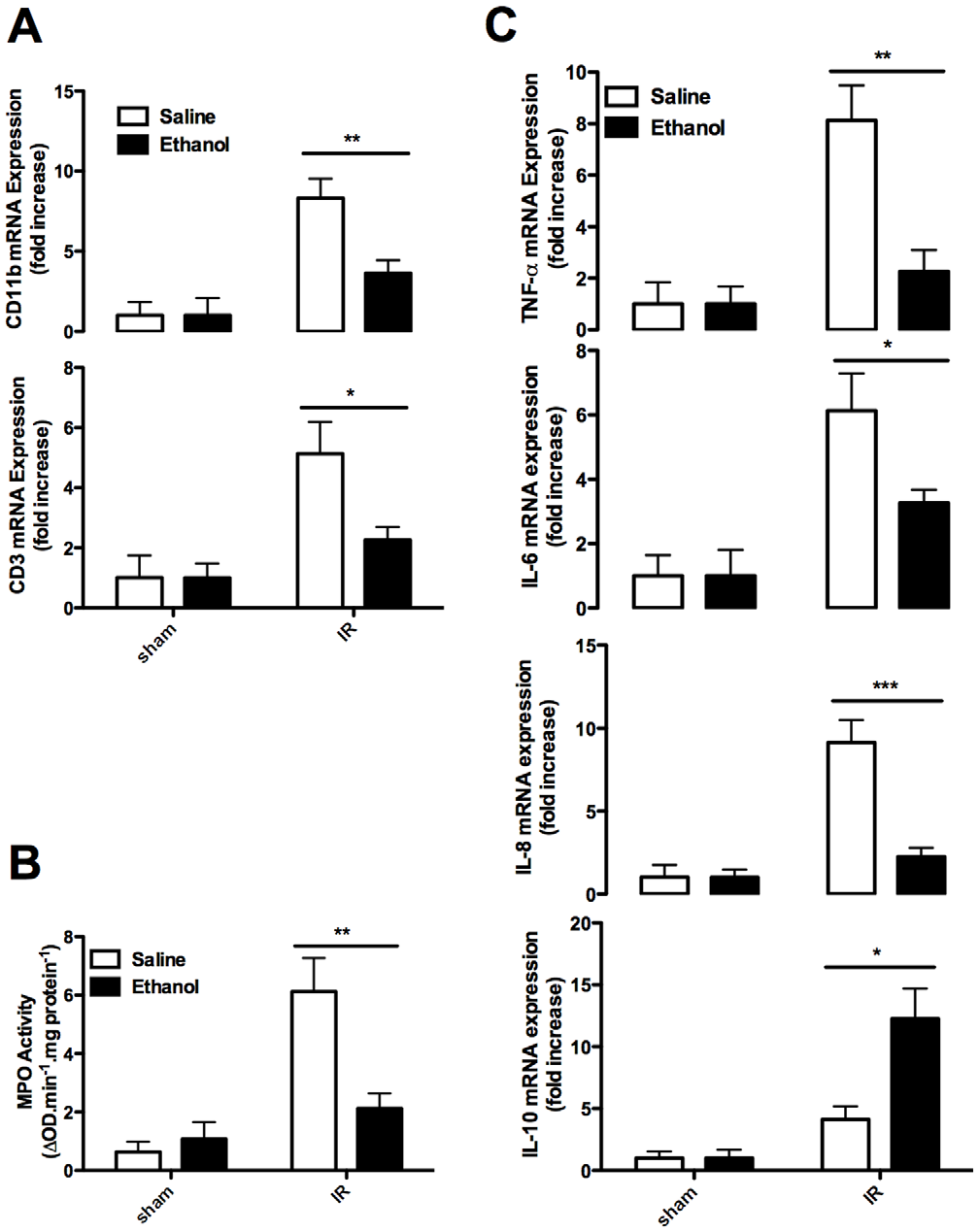
Ethanol pretreatment attenuated inflammation after renal IR. Mice were pretreated with saline or 1 g/kg ethanol, and were exposed to a 30-min bilateral renal IR 3 h later. Kidney samples were collected at 24 h post reperfusion and assessed for: **A.** CD11b, CD3 mRNA expression; **B.** MPO activity; and **C.** TNF-α, IL-6, IL-8 and IL-10 mRNA expression. Results are mean values ± SEM. (*n* = 4–6). (* *p*<0.05, ** *p*<0.01, *** *p*<0.001).

We then assessed the intra kidney inflammatory infiltration by RT-PCR analysis. We first examined CD11b and CD3 expression, the two critical markers for myeloid cells and T cells, respectively. It was noted that CD11b and CD3 were significantly increased in the kidneys 24 h after reperfusion. On the other hand, Pretreatment of mice with 1 g/kg of ethanol reduced CD11b mRNA by 56.3% and CD3 mRNA by 55.9% (Figure 4A). Next, we performed MPO assay to assess the severity of neutrophil infiltration, and similar results were obtained (Figure 4B). Given the fact that cytokines such as TNF-α, IL-6, IL-8 and IL-10 play critical roles in IR-induced renal injury, we therefore further compared their expressions between ethanol treated mice and control mice. Interestingly, mRNAs for TNF-α, IL-6 and IL-8 were significantly higher in control mice as compared with that of ethanol treated mice. In sharp contrast, IL-10 was significantly higher in ethanol treated mice 24 h after IR induction (Figure 4C).

### 3. Ethanol pretreatment decreased IR-induced oxidative stress in the kidneys

**Figure 5:**
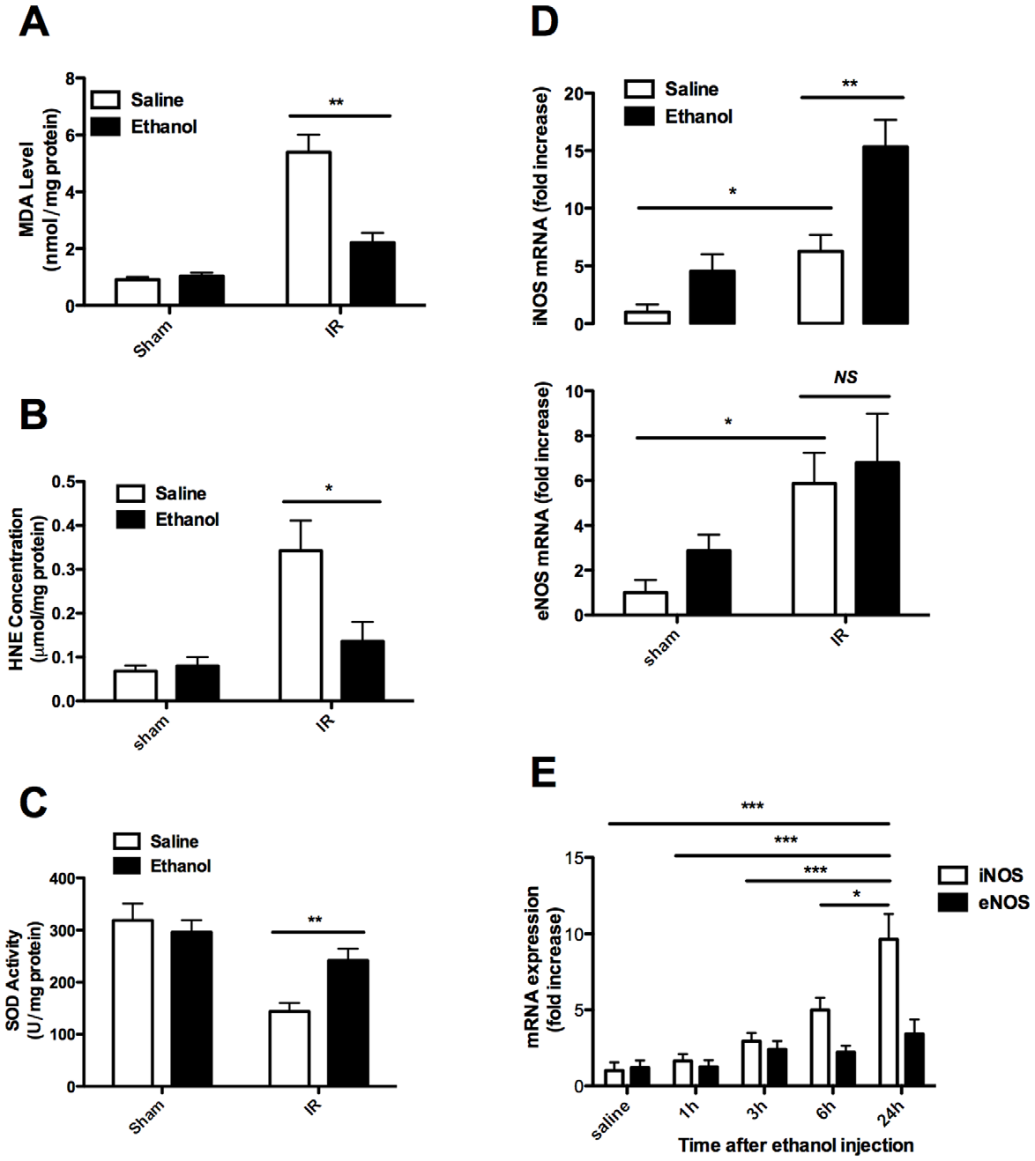
Ethanol pretreatment ameliorated oxidative stress after renal IR. Mice were pretreated with saline or 1 g/kg ethanol, and were exposed to a 30-min bilateral renal IR 3 h later. Kidney samples were collected at 24 h post reperfusion for assay of: **A.** MDA levels; **B.** HNE levels; and **C.** SOD activities; and **D.** iNOS & eNOS expression. **E.** Mice were pretreated with saline or ethanol (1 g/kg) and exposed to a 30-min bilateral renal ischemia after different time interval. Kidney samples were collected at 24 h post reperfusion for assay of iNOS & eNOS expression. Results are mean values ± SEM. (*n* = 4–6). (* *p*<0.05, ** *p*<0.01).

To dissect the underlying mechanisms by which antecedent ethanol exposure protects kidneys against IR-induced injury, we first examined oxidative stress in the kidneys of mice treated with either ethanol or saline by measuring MDA production and HNE concentration. It was interestingly noted that the production of MDA and the concentration of HNE in the kidneys originated from mice with antecedent ethanol exposure were significantly lower as compared with that of control mice (Figure 5A & 5B), demonstrating that antecedent ethanol exposure inhibits IR-induced oxidative stress in the kidneys. To demonstrate whether the reduced oxidative stress relevant to ethanol pretreatment is associated with enhanced antioxidant capacity, we next examined SOD activity, the critical enzyme responsible for detoxifying ROS. As expected, kidneys originated from mice with antecedent ethanol exposure showed significantly higher SOD activity after IR induction as compared with that of control mice (Figure 5C), suggesting that ethanol pretreatment enhanced antioxidant capability. We further determined the expression of inducible nitric oxide synthase (iNOS) and endothelial nitric oxide synthase (eNOS) in the kidneys after IR insult. As shown in Figure 5D, both iNOS and eNOS were induced 24 h post reperfusion. However, ethanol pretreatment enhanced much higher iNOS expression as compared with that of controls. Kinetic studies revealed that the potency for inducing iNOS expression was much stronger at 24 h post reperfusion than that of earlier time points such as 1–6 h after ethanol exposure (Figure 5E).

### 4. Ethanol pretreatment enhanced ALDH2 activity to prevent IR-induced lipid peroxidation

**Figure 6:**
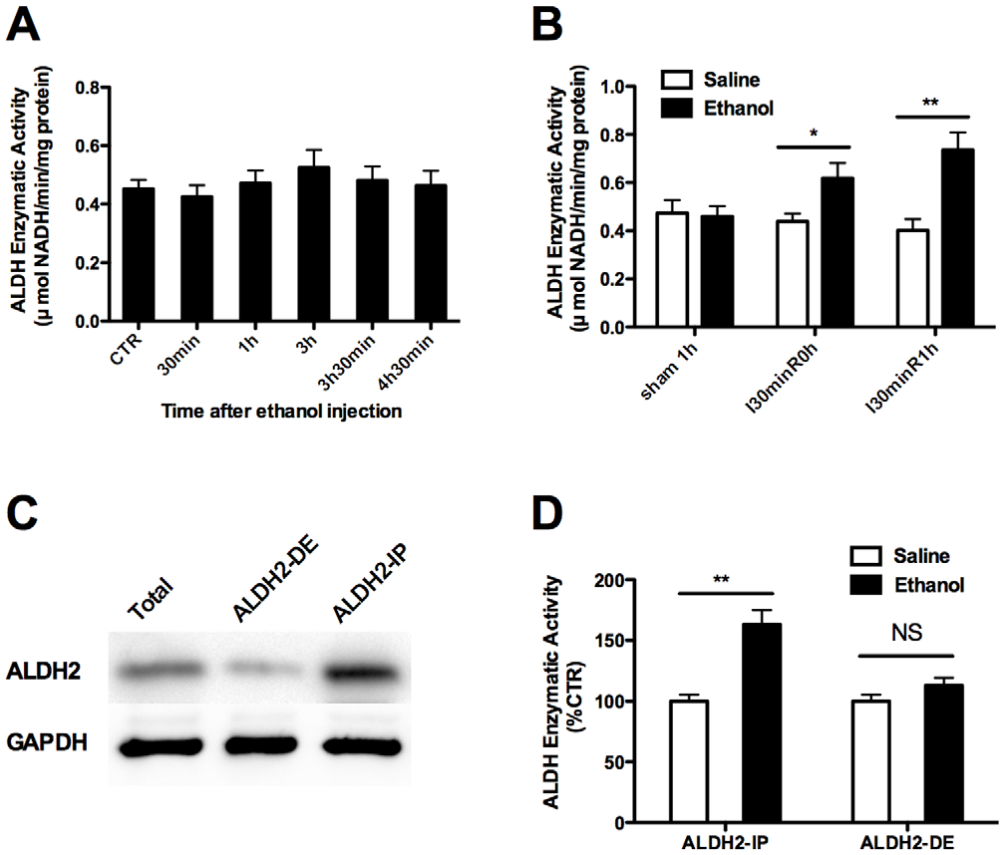
Ethanol pretreatment increased ALDH2 enzymatic activity after renal IR. **A.** Mice were pretreated with 1 g/kg ethanol and kidney samples were collected for ALDH enzymatic activity assay at different time after ethanol exposure. The CTR group did not receive any ethanol. **B.** Mice were pretreated with saline or 1 g/kg ethanol, and were exposed to a 30-min bilateral renal IR 3 h later. Kidney samples were collected at different time after reperfusion. **C.** Immunoprecipitation was performed to separate the total protein from kidney tissues to the ALDH2-enriched part (ALDH2-IP), and the ALDH2-depleted part (ALDH2-DE). **D.** ALDH enzymatic assay was performed in the ALDH2 enriched or depleted proteins. Results are mean values ± SEM. (*n* = 4–5). (* *p*<0.05, ** *p*<0.01).

As lipid peroxidation due to excessive generation of MDA and HNE plays a critical role in IR-induced renal injury, the above results prompted us to further examine the impact of antecedent ethanol exposure on lipid peroxidation after IR induction. For this purpose, we checked the enzymatic activity of ALDH, a family of metabolic enzymes involved in both ethanol metabolism and reactive aldehydes such as HNE detoxification. It was noted that ethanol administration itself did not show a significant impact on ALDH activities (Figure 6A). However, when mice were pretreated with ethanol, the ALDH activities were significantly higher upon IR induction, and a 1 - fold increase was observed 1 h after reperfusion (Figure 6B). Given the fact that kidneys express multiple ALDH isozymes such as ALDH2, ALDH3a1, ALDH3a2, ALDH4a1, ALDH7a1 and ALDH8a1, we tried to document changes in gene transcription and protein expression for each isozyme, but failed to get any significant results between saline and ethanol pretreated mice (data not shown). As ALDH2 has been proved to be a key metabolic enzyme involved in both ethanol metabolism and the detoxification of other reactive aldehydes such as HNE, we next sought to determine whether ALDH2 plays a predominant role of the protection induced by ethanol pretreatment. To this end, we performed immunoprecipitation with antibody against ALDH2 to separate the total protein from renal lysates into the ALDH2-enriched immunoprecipitates, and the residual ALDH2-depleted part. In line with our expectation, ALDH activities were significantly higher in ethanol pretreated mice as compared with control mice in ALDH2-enriched immunoprecipitates resulted from the same amount of renal lysates. Particularly, when ALDH2 was depleted, both ethanol pretreated and control mice showed similar ALDH activities (Figure 6C & 6D). Altogether, our results suggest that antecedent ethanol exposure before IR insult increases ALDH2 activation, which then protects kidneys against IR-induced lipid peroxidation.

### 5. Knock-down of ALDH2 expression abrogated the protective effect of ethanol pretreatment on renal IR injury

**Figure 7:**
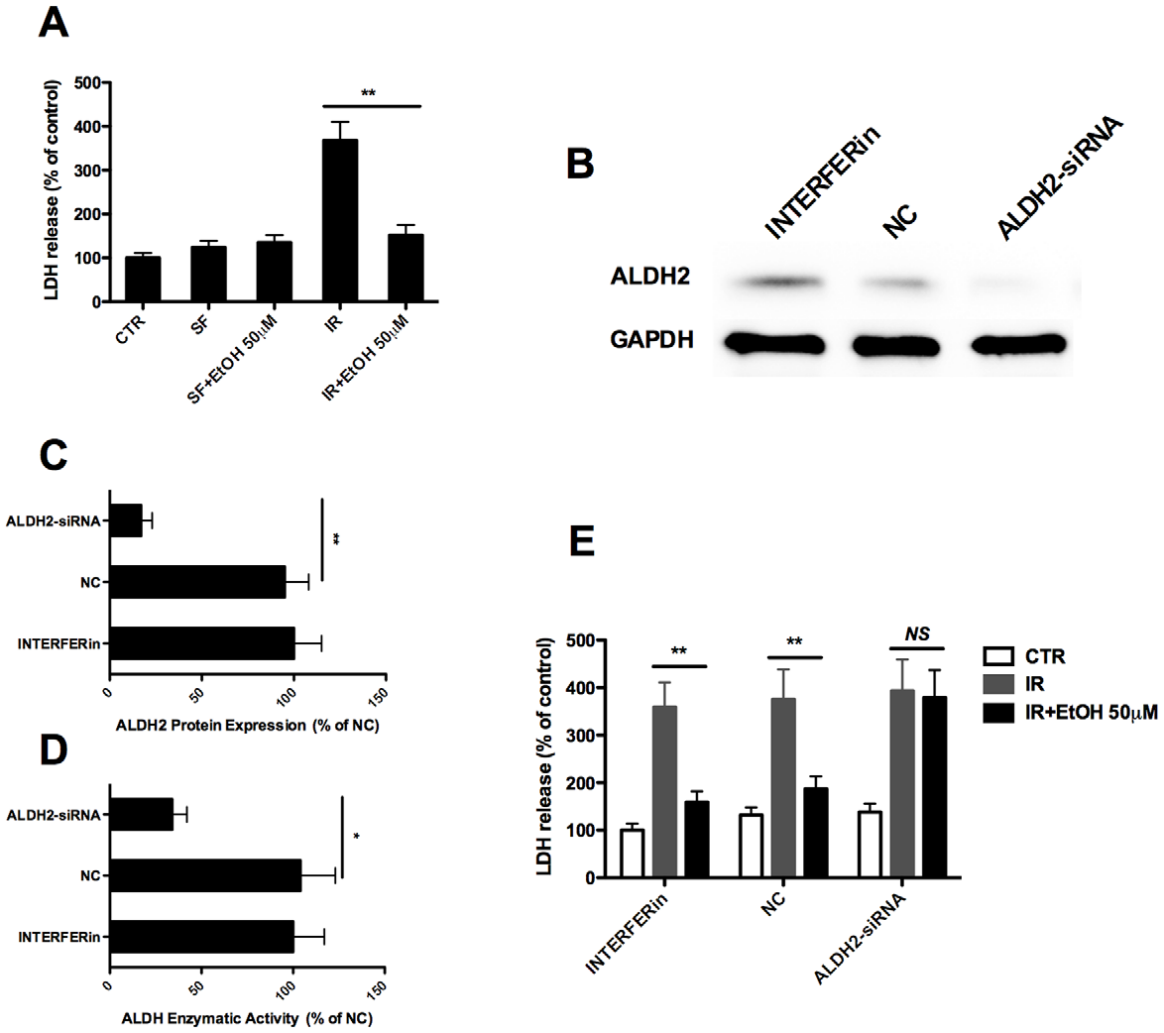
The protection induced by ethanol pretreatment is ALDH2 dependent. **A.** Primary cultured TECs were starved in serum-free (SF) medium for 24 h. Simulated IR were induced by immersing cell monolayer in mineral oil for 60 min at 37°C, then incubated in full medium for another 24 h and assayed the LDH release. Ethanol pretreatment were given at 3 h before ischemia at a dose of 50 µM. **B.** and **C.** Primary cultured TECs were transfected with negative control siRNA (NC) or ALDH2-siRNA using INTERFERin™ transfection reagent. The INTERFERin group served as the empty control. ALDH2 protein contents were assayed by western blot at 48 h post transfection. **D.** Cells were treated as in **B**, and ALDH enzymatic activities were assessed at 48 h post transfection. **E.** Cells were treated as in **B**, and then induced simulated IR as in **A** at 48 h post transfection. LDH release was assayed at 24 h post transfection. Results are mean values ± SEM. (*n* = 4–6). (* *p*<0.05, ** *p*<0.01).

To further confirm the above results, we employed an *in vitro* simulation IR model in primary renal TECs. We first confirmed that ethanol pretreatment (50 µM) of TECs 3 h before simulated IR reduced LDH release (Figure 7A). Next, an ALDH2 specific siRNA was transfected into the primary renal TECs, which resulted in an 82.1% decrease for ALDH2 protein levels 48 h after transfection as manifested by Western blotting (Figure 7B) followed by densitometric analysis (Figure 7C). Consistently, the ALDH enzymatic activities were decreased by 67.3% (Figure 7D). Importantly, when the same protocol of ethanol pretreatment and simulated IR was applied in the ALDH2 siRNA transfected cells, the protective effect induced by ethanol pretreatment was almost completely abrogated (Figure 7E).

## Discussion

Although the protective effect for consumption of alcoholic beverages at low to moderate levels (1–3 drinks/day for months to years) have been widely documented in cardiovascular diseases [33], [34], [35], less investigation is done in its relation to the kidney diseases [36]. Previous studies revealed that long-term alcohol abuse is associated with many renal alterations in humans [37], but moderate amount of red wine for diabetic patients is suggested to be helpful for slowing down the progression of diabetic nephropathy [38]. Experimental studies paid more attention to the polyphenols in the wine and found that they could enhance renal antioxidant defense [39], [40] and inhibit mesangial cell apoptosis [41], [42], through which red wines were suggested to protect kidneys against IR injury [43], [44]. The exact effect of ethanol on renal IR injury, however, remains poorly elucidated [36].

The present studies for the first time demonstrated that moderate ethanol administration antecedent to renal IR induction provides protection in a dose and time dependent manner. After bilateral renal ischemia, ethanol pretreated mice displayed significantly preserved renal function as characterized by less histological tubular damage, reduced leukocyte infiltration and proinflammatory cytokine production. We further demonstrated that the protective effect was associated with ameliorated oxidative stress as manifested by the increase of antioxidant capacity (higher SOD activities) and decrease of lipid peroxidation (less generation of MAD and HNE). Further studies revealed that ethanol administration antecedent to IR insult significantly enhanced the enzymatic activity of ALDH2, which then promoted the detoxification of aldehydic metabolites to prevent lipid peroxidation. By employing an in vitro IR simulation model, we confirmed that the protective effect of antecedent ethanol exposure on IR-induced renal injury dependent on the induction of ALDH2. Transfection of a siRNA specific for ALDH2 into the primary cultured TECs completely abrogated the protective effect. Taken together, our data demonstrate that moderate antecedent ethanol exposure provides protection for kidneys against subsequent IR-induced injury by increasing antioxidant capacity and by preventing lipid peroxidation through increasing the activity of ALDH2.

Our data presented in the current report are in agreement with previous studies in the heart, intestine and brain after IR induction [16], [17], [19], [20], [45]. Using an intestine IR model, Yamaguchi et al. demonstrated that single bolus ethanol ingestion markedly attenuated IR induced increases in leukocyte rolling and adhesion [20]. They reported a biphasic temporal pattern of protection against the proinflammatory effects, which included the early phase at 2–3 h and the late phase at 24 h post ethanol pretreatment, and suggested that the late phase of ethanol pretreatment is triggered by NO formed secondary to adenosine A2 receptor-dependent activation of iNOS during the period of ethanol exposure. In another brain IR injury model, a late phase (24 h) protection against neurodegenerative processes was also observed in moderate ethanol ingested mice, and an NADPH oxidase dependent triggering mechanism was reported to be critically involved [19].

Interestingly, we observed a similar biphasic protection against renal IR injury in ethanol antecedent exposed mice, but more powerful in the early phase at 3 h after ethanol pretreatment. We ruled out the possibility that differences in approaches for ethanol administration might affect the results, as we observed almost the same peak levels of ethanol in the blood and same elimination kinetics as compare with that in precious studies [19], [20]. Similar to the studies of LPS pretreatment [46], we found that ethanol pretreatment enhanced iNOS rather than eNOS expression after IR insult. Our kinetic studies further suggest that the capacity to enhance iNOS expression at 24 h post reperfusion was higher at the late phase (i.e.,24 h) in comparison with that at the earlier phases (e.g.,1–6 h) after ethanol pretreatment. These results suggest that the enhanced iNOS expression may be responsible for the late phase protection conferred by ethanol pretreatment. However, further studies using iNOS or eNOS deficient mice would be necessary to elucidate the exact impact of the NOS-NO system on ethanol-induced protection in renal IR injury.

The early phase (60 min) effect of ethanol pretreatment was extensively studied in the cardiac IR model [17]. The predominant role in mediating cytoprotection was suggested to be attributed to ALDH2, an intra-mitochondrial enzyme involved in the detoxification of toxic aldehydes such as HNE [16], [29]. In line with this result, we found that the early phase protection in our model was also associated with markedly elevated ALDH enzymatic activity in the kidneys. Our *in vitro* studies demonstrated that the early phase protection was ALDH2 dependent since knockdown of ALDH2 by siRNA abrogated the protective effect.

To rule out that direct chemical stimulation due to high concentration of ethanol might affect the results, we kept constant ethanol concentration at 5%. Therefore, the total saline volumes among each ethanol dose groups were different, and the control group received the largest volume of saline as that in the 2 g/kg body weight dose group. Nevertheless, the volumes of saline before kidney IR induction did not show a perceptible effect on the response to IR injury, as we failed to observe any difference on the renal function after IR insult between the mice injected with or without saline at the largest volume (data not shown). Despite that better renal function was observed based on the histological results in the kidneys of ethanol-pretreated mice at day 7 post reperfusion, we cannot exclude the possibility that the well reserved renal function was due to that ethanol treatment accelerated the recovery of renal function after IR insult. As such, further studies would be necessary with focus on to verify whether ethanol pretreatment accelerates renal functional recovery. It is noteworthy that pretreatment with high dose of ethanol (2 g/Kg) failed to protect the animals against IR injury. Given our observations that the predominant protective effect occurs in the short-term period (i.e., 3 h), the protective effect for the high dose of ethanol could be overwhelmed by its damaging impact on the kidneys that concomitantly occurs within the same window of time period. As a consequence, additional mechanistic studies would be important to clarify the narrow therapeutic window for ethanol pretreatment in the prevention of IR-induced renal injury.

In summary, our data resulted from a renal IR model provide the first line of experimental evidence for the effect of antecedent ethanol exposure by single bolus *i.p.* injection on biphasic protection of kidneys against IR-induced injury, with much stronger protective effect observed 3 h after ethanol administration (early phase). Moreover, our work suggests that the protective effect is ALDH2 dependent, which serves as a key enzyme in eliminating the toxic aldehydes produced by IR to prevent lipid peroxidation. Altogether, our results demonstrate that moderate ethanol ingestion and ectopic ALDH2 activation could be viable therapeutic approaches for various clinical conditions involving renal IR injury.

## References

[1] Gelman S (1995). The pathophysiology of aortic cross-clamping and unclamping.. Anesthesiology.

[2] Chertow GM, Burdick E, Honour M, Bonventre JV, Bates DW (2005). Acute kidney injury, mortality, length of stay, and costs in hospitalized patients.. J Am Soc Nephrol.

[3] Pomfret EA, Sung RS, Allan J, Kinkhabwala M, Melancon JK (2008). Solving the organ shortage crisis: the 7th annual American Society of Transplant Surgeons' State-of-the-Art Winter Symposium.. Am J Transplant.

[4] Sheridan AM, Bonventre JV (2000). Cell biology and molecular mechanisms of injury in ischemic acute renal failure.. Curr Opin Nephrol Hypertens.

[5] Bonventre JV, Zuk A (2004). Ischemic acute renal failure: an inflammatory disease?. Kidney Int.

[6] Akcay A, Nguyen Q, Edelstein CL (2009). Mediators of inflammation in acute kidney injury.. Mediators Inflamm.

[7] Kinsey GR, Li L, Okusa MD (2008). Inflammation in acute kidney injury.. Nephron Exp Nephrol.

[8] Hayama T, Matsuyama M, Funao K, Tanaka T, Tsuchida K (2007). Triggers of inflammation after renal ischemia/reperfusion.. Transplant Proc.

[9] Noiri E, Nakao A, Uchida K, Tsukahara H, Ohno M (2001). Oxidative and nitrosative stress in acute renal ischemia.. Am J Physiol Renal Physiol.

[10] Walker LM, York JL, Imam SZ, Ali SF, Muldrew KL (2001). Oxidative stress and reactive nitrogen species generation during renal ischemia.. Toxicol Sci.

[11] Lloberas N, Torras J, Herrero-Fresneda I, Cruzado JM, Riera M (2002). Postischemic renal oxidative stress induces inflammatory response through PAF and oxidized phospholipids. Prevention by antioxidant treatment.. Faseb J.

[12] Aragno M, Cutrin JC, Mastrocola R, Perrelli MG, Restivo F (2003). Oxidative stress and kidney dysfunction due to ischemia/reperfusion in rat: attenuation by dehydroepiandrosterone.. Kidney Int.

[13] McCord JM (1985). Oxygen-derived free radicals in postischemic tissue injury.. N Engl J Med.

[14] Eschwege P, Paradis V, Conti M, Holstege A, Richet F (1999). In situ detection of lipid peroxidation by-products as markers of renal ischemia injuries in rat kidneys.. J Urol.

[15] Niki E, Yoshida Y, Saito Y, Noguchi N (2005). Lipid peroxidation: mechanisms, inhibition, and biological effects.. Biochem Biophys Res Commun.

[16] Chen CH, Budas GR, Churchill EN, Disatnik MH, Hurley TD (2008). Activation of aldehyde dehydrogenase-2 reduces ischemic damage to the heart.. Science.

[17] Churchill EN, Disatnik MH, Mochly-Rosen D (2009). Time-dependent and ethanol-induced cardiac protection from ischemia mediated by mitochondrial translocation of varepsilonPKC and activation of aldehyde dehydrogenase 2.. J Mol Cell Cardiol.

[18] Vasiliou V, Nebert DW (2005). Analysis and update of the human aldehyde dehydrogenase (ALDH) gene family.. Hum Genomics.

[19] Wang Q, Sun AY, Simonyi A, Kalogeris TJ, Miller DK (2007). Ethanol preconditioning protects against ischemia/reperfusion-induced brain damage: role of NADPH oxidase-derived ROS.. Free Radic Biol Med.

[20] Yamaguchi T, Dayton C, Shigematsu T, Carter P, Yoshikawa T (2002). Preconditioning with ethanol prevents postischemic leukocyte-endothelial cell adhesive interactions.. Am J Physiol Heart Circ Physiol.

[21] Yamaguchi T, Dayton CB, Ross CR, Yoshikawa T, Gute DC (2003). Late preconditioning by ethanol is initiated via an oxidant-dependent signaling pathway.. Free Radic Biol Med.

[22] Wuthrich RP, Glimcher LH, Yui MA, Jevnikar AM, Dumas SE (1990). MHC class II, antigen presentation and tumor necrosis factor in renal tubular epithelial cells.. Kidney Int.

[23] Wu H, Chen G, Wyburn KR, Yin J, Bertolino P (2007). TLR4 activation mediates kidney ischemia/reperfusion injury.. J Clin Invest.

[24] Meldrum KK, Meldrum DR, Hile KL, Burnett AL, Harken AH (2001). A novel model of ischemia in renal tubular cells which closely parallels in vivo injury.. J Surg Res.

[25] Kim J, Jang HS, Park KM (2010). Reactive oxygen species generated by renal ischemia and reperfusion trigger protection against subsequent renal ischemia and reperfusion injury in mice.. Am J Physiol Renal Physiol.

[26] Kim KH, Dhupar R, Ueki S, Cardinal J, Pan P (2009). Donor graft interferon regulatory factor-1 gene transfer worsens liver transplant ischemia/reperfusion injury.. Surgery.

[27] Esterbauer H, Koller E, Slee RG, Koster JF (1986). Possible involvement of the lipid-peroxidation product 4-hydroxynonenal in the formation of fluorescent chromolipids.. Biochem J.

[28] Hart ML, Much C, Kohler D, Schittenhelm J, Gorzolla IC (2008). Use of a hanging-weight system for liver ischemic preconditioning in mice.. Am J Physiol Gastrointest Liver Physiol.

[29] Budas GR, Disatnik MH, Chen CH, Mochly-Rosen D Activation of aldehyde dehydrogenase 2 (ALDH2) confers cardioprotection in protein kinase C epsilon (PKCvarepsilon) knockout mice.. J Mol Cell Cardiol.

[30] Castilla R, Gonzalez R, Fouad D, Fraga E, Muntane J (2004). Dual effect of ethanol on cell death in primary culture of human and rat hepatocytes.. Alcohol Alcohol.

[31] de Labry LO, Glynn RJ, Levenson MR, Hermos JA, LoCastro JS (1992). Alcohol consumption and mortality in an American male population: recovering the U-shaped curve–findings from the normative Aging Study.. J Stud Alcohol.

[32] Fuchs CS, Stampfer MJ, Colditz GA, Giovannucci EL, Manson JE (1995). Alcohol consumption and mortality among women.. N Engl J Med.

[33] Gaziano JM, Gaziano TA, Glynn RJ, Sesso HD, Ajani UA (2000). Light-to-moderate alcohol consumption and mortality in the Physicians' Health Study enrollment cohort.. J Am Coll Cardiol.

[34] Rimm EB, Giovannucci EL, Willett WC, Colditz GA, Ascherio A (1991). Prospective study of alcohol consumption and risk of coronary disease in men.. Lancet.

[35] Pearson TA (1996). Alcohol and heart disease.. Circulation.

[36] Presti RL, Carollo C, Caimi G (2007). Wine consumption and renal diseases: new perspectives.. Nutrition.

[37] Cecchin E, De Marchi S (1996). Alcohol misuse and renal damage.. Addict Biol.

[38] Facchini FS, Saylor KL (2003). A low-iron-available, polyphenol-enriched, carbohydrate-restricted diet to slow progression of diabetic nephropathy.. Diabetes.

[39] Krajka-Kuzniak V, Baer-Dubowska W (2003). The effects of tannic acid on cytochrome P450 and phase II enzymes in mouse liver and kidney.. Toxicol Lett.

[40] Satyanarayana PS, Singh D, Chopra K (2001). Quercetin, a bioflavonoid, protects against oxidative stress-related renal dysfunction by cyclosporine in rats.. Methods Find Exp Clin Pharmacol.

[41] Li X, Kimura H, Hirota K, Kasuno K, Torii K (2005). Synergistic effect of hypoxia and TNF-alpha on production of PAI-1 in human proximal renal tubular cells.. Kidney Int.

[42] Ishikawa Y, Kitamura M (2000). Bioflavonoid quercetin inhibits mitosis and apoptosis of glomerular cells in vitro and in vivo.. Biochem Biophys Res Commun.

[43] Shigematsu S, Ishida S, Hara M, Takahashi N, Yoshimatsu H (2003). Resveratrol, a red wine constituent polyphenol, prevents superoxide-dependent inflammatory responses induced by ischemia/reperfusion, platelet-activating factor, or oxidants.. Free Radic Biol Med.

[44] Kahraman A, Erkasap N, Serteser M, Koken T (2003). Protective effect of quercetin on renal ischemia/reperfusion injury in rats.. J Nephrol.

[45] Dayton C, Yamaguchi T, Kamada K, Carter P, Korthuis RJ (2004). Antecedent ethanol ingestion prevents postischemic P-selectin expression in murine small intestine.. Microcirculation.

[46] Kim JI, Jang HS, Park KM (2010). Endotoxin-induced renal tolerance against ischemia and reperfusion injury is removed by iNOS, but not eNOS, gene-deletion.. BMB reports.

